# Inhibiting nuclear factor erythroid 2 related factor 2-mediated autophagy in bovine mammary epithelial cells induces oxidative stress in response to exogenous fatty acids

**DOI:** 10.1186/s40104-022-00695-2

**Published:** 2022-04-10

**Authors:** Renxu Chang, Xudong Sun, Hongdou Jia, Qiushi Xu, Zhihao Dong, Yan Tang, Shengbin Luo, Qianming Jiang, Juan J. Loor, Chuang Xu

**Affiliations:** 1 College of Veterinary Medicine, China Agricultural University, Yuan Ming Yuan West Road No. 2, Haidian District, Beijing, 100193 China; 2 College of Veterinary Medicine, Hunan Agricultural University, Changsha, 410128 China; 3 Heilongjiang Provincial Key Laboratory of Prevention and Control of Bovine Diseases, College of Animal Science and Veterinary Medicine, Heilongjiang Bayi Agricultural University, No. 5 Xinyang Road, Daqing, 163319 Heilongjiang Province China; 4 Mammalian NutriPhysioGenomics, Department of Animal Sciences and Division of Nutritional Sciences, University of Illinois, Urbana, 61801 USA

**Keywords:** Autophagy, Bovine mammary epithelial cells, NFE2L2, Oxidative stress

## Abstract

**Background:**

In early lactation, bovine mammary epithelial cells undergo serious metabolic challenges and oxidative stress both of which could be alleviated by activation of autophagy. Nuclear factor erythroid 2 related factor 2 (NFE2L2), a master regulator of cellular redox homeostasis, plays an important role in the regulation of autophagy and oxidative stress. Thus, the objective of this study was to investigate the role of NFE2L2-mediated autophagy on oxidative stress of bovine mammary epithelial cells in response to exogenous free fatty acids (FFA).

**Results:**

Exogenous FFA induced linear and quadratic decreases in activities of glutathione peroxidase (GSH-Px), catalase (CAT), and superoxide dismutase (SOD), and increases in the contents of reactive oxygen species (ROS) and malondialdehyde (MDA). Protein abundance of LC3-phosphatidylethanolamine conjugate (LC3-II) and the number of autophagosomes and autolysosomes decreased in a dose-dependent manner, while protein abundance of p62 increased in cells challenged with FFA. Activation of autophagy via pre-treatment with Rap attenuated the FFA-induced ROS accumulation. Importantly, FFA inhibited protein abundance of NFE2L2 and the translocation of NFE2L2 into the nucleus. Knockdown of *NFE2L2* by siRNA decreased protein abundance of LC3-II, while it increased protein abundance of p62. Furthermore, sulforaphane (SFN) pre-treatment attenuated the FFA-induced oxidative stress by activating NFE2L2-mediated autophagy.

**Conclusions:**

The data suggested that NFE2L2-mediated autophagy is an important antioxidant mechanism in bovine mammary epithelial cells experiencing increased FFA loads.

## Introduction

The rapid increase in milk production coupled with insufficient energy intake in early lactation drives cows into a period of negative energy balance [1]. Severe negative energy balance increases circulating concentrations of free fatty acids (FFA) by initiating fat mobilization of adipose tissue [2], which render bovine mammary gland highly-susceptible to metabolic stress [2, 3]. In cows experiencing ketosis after calving, the high concentrations of FFA also are absorbed by the mammary gland and augments the metabolic stress within mammary cells [4, 5]. High concentrations of FFA induce mitochondrial dysfunction and ROS within mammary epithelial cells and causes oxidative stress [5, 6].

Autophagy is a conserved degradation process, which plays a cardinal role in the maintenance of cellular homeostasis via eliminating damaged cellular organelles and scavenging aging proteins [7–9]. Activation of autophagy, a major cellular defense process, alleviated metabolic stress and its associated pro-oxidant state in non-ruminants [10, 11]. More importantly, work in dairy cows has suggested that enhanced autophagy is an adaptive mechanism to help mitigate metabolic stress in the mammary gland induced by hyperketonemia [5]. In vitro, activation of autophagy protected bovine mammary epithelial cells against H_2_O_2_-induced oxidative stress and apoptosis [4]. Thus, mitigation of oxidative challenges via autophagy might be an important defense mechanism in bovine mammary epithelial cells.

Nuclear factor erythroid 2 related factor 2 (NFE2L2) is a master transcription factor, which maintains redox state in the cell through regulating the transcription of numerous antioxidants [12–14]. Activation of NFE2L2 is essential to protect bovine mammary epithelial cells against H_2_O_2_-induced oxidative stress [15]. In addition, NFE2L2 is also a regulator of autophagy [13, 16]. Work with non-ruminants confirmed that NFE2L2 activates autophagy through the control of LAMP2A transcription, which alleviates oxidative stress by inhibiting accumulation of oxidized protein aggregates [17]. Interestingly, the regulation of autophagy via NFE2L2 also was confirmed in primary bovine mammary epithelial cells [18]. Thus, NFE2L2-mediated autophagy might play a protective role against oxidative stress in bovine mammary epithelial cells.

Given the established role of NEF2L2 in the regulation of autophagy and oxidative stress in non-ruminants, we hypothesized that activation of NEF2L2-mediated autophagy protects bovine mammary epithelial cells against oxidative stress. In the present study, sulforaphane (SFN) and NFE2L2 small interfering RNA (Si-NFE2L2) were used to upregulate and downregulate NFE2L2 abundance in MAC-T cells. The main objective was to investigate effects of NFE2L2-mediated autophagy on oxidative stress in bovine mammary epithelial cells in response to exogenous FFA.

## Materials and methods

### Cell culture and treatments

The bovine mammary epithelial cell line (MAC-T) was purchased from Shanghai chuanqiu Biotechnology Co., Ltd. and was cultured in Dulbecco’s modified Eagle medium/F-12 medium (DMEM/F-12, Grand Island, New York, USA) supplemented with 10 μg/L insulin (Sigma-Aldrich, St. Louis, MO, USA), 10% fetal bovine serum (Hyclone, Grand Island, New York, USA), 100 U/mL penicillin (Hyclone, South Logan, UT, USA), 100 μg/mL streptomycin (Hyclone, South Logan, UT, USA), and growth-promoting hormones (1 mg/L progesterone, 0.05% lactalbumin, and 0.05% α-lactose) in an incubator at 37 °C incubator with 5% CO_2_. The medium was changed every 24 h. When cell density reached 80%, cells were detached with trypsin, and incubated at 37 °C for 5 min before passage into new cell culture flasks. The lactogenic medium (DMEM/F-12 supplemented with 1 g/L BSA and 2.5 mg/L prolactin) was used to culture MAC-T cells before each treatment [19].

In Exp.1, to investigate the effects of FFA on oxidative stress and autophagy, different FFA concentrations were chosen to treat MAC-T cells to simulate the response observed in ketotic cows after calving [6, 20]. The MAC-T cells were cultured in 2% fatty acid-free BSA with 0, 0.6, 1.2, or 2.4 mmol/L FFA for 24 h. Cells were cultured in serum-free medium for 12 h before the above treatments to induce autophagy as previous described [21, 22]. The stock FFA mix was prepared as previously described [23]: palmitic acid (16.8 mmol/L), stearic acid (7.6 mmol/L), oleic acid (22.9 mmol/L), linoleic acid (2.6 mmol/L), and palmitoleic acid (2.8 mmol/L) were diluted in 0.1 mol/L KOH solution at 60 °C, and the pH of the was adjusted to 7.4 with 1 mol/L hydrochloric acid.

In Exp.2, to investigate the effect of autophagy activation on FFA-induced oxidative stress, MAC-T cells were seeded into 6-well plates with lactogenic medium and grown until 80% to 90% confluence. Rapamycin (Rap) was diluted in dimethylsulfoxide (DMSO) to a final working concentration. There were 4 experimental treatments: BSA + DMSO, DMSO was used to treat cells for 18 h prior to addition of 2% BSA for an additional 24 h; 1.2 mmol/L FFA + DMSO, DMSO was used to treat cells for 18 h prior to challenge with 1.2 mmol/L FFA for 24 h; BSA + 100 nmol/L Rap, 100 nmol/L Rap was used to treat cells for 18 h prior to addition of 2% BSA for an additional 24 h; FFA + 100 nmol/L Rap, 100 nmol/L Rap was used to treat cells for 18 h prior to challenge with 1.2 mmol/L FFA for an additional 24 h.

In Exp.4, to investigate the role of NFE2L2 activation on autophagy and oxidative stress in MAC-T induced by FFA, cells were seeded into 6-well plates with lactogenic medium and grown until 80% to 90% confluence. SFN was diluted in DMSO to a final working concentration. The 4 experimental treatments included the following: BSA + DMSO, DMSO was used to treat cells for 24 h prior to addition of 2% BSA for an additional 24 h; 1.2 mmol/L FFA + DMSO, DMSO was used to treat cells for 24 h prior to challenge with 1.2 mmol/L FFA for 24 h; BSA + 10 μmol/L SFN, 10 μmol/L SFN was used to treat cells for 24 h prior to addition of 2% BSA for an additional 24 h; FFA + 10 μmol/L SFN, 10 μmol/L SFN was used to treat cells for 24 h prior to challenge with 1.2 mmol/L FFA for an additional 24 h.

### Adenovirus transfections

The mRFP-GFP-LC3 adenoviral vectors (Ad-mRFP-GFP-LC3, 1 × 10^10^ plaque-forming units/mL) were purchased from Hanbio (HB-AP2100001, Shanghai, China). Before treatment with LC3 adenovirus, cells were seeded in 6-well plates (2.0 × 10^6^ cells/cm^2^) and cultured in lactogenic medium at 37 °C with 5% CO_2_. When cell density reached 30–50%, cells were transfected with 50 multiplicities of infection of adenovirus for 6 h in DMEM/F-12 medium without serum and antibiotic. Subsequently, the medium was switched to DMEM/F12 medium supplemented containing 10% fetal bovine serum, 100 U/mL penicillin, and 100 μg/mL streptomycin for 42 h. Cells were then treated with or without 1.2 mmol/L FFA for 24 h in 2% BSA medium. Cells were washed 3 times with phosphate-buffered saline (PBS) before being fixed with 4% paraformaldehyde for 30 min at room temperature. Cells were then stained with DAPI (10 μg/mL) (D8417, Sigma-Aldrich) at room temperature for 10 min. After washing 3 times with PBS, the image was collected with a laser confocal microscope (Fluoview FV1200, Olympus, Tokyo, Japan).

### RNA interference

In Exp.3, to explore the effect of NFE2L2 knockdown on autophagy in MAC-T cells, scrambled non-target negative control (Si-Control) or siRNA targeting NFE2L2 (Si-NFE2L2) were used to transfect MAC-T cells for 48 h. The Si-NFE2L2 and Si-Control were designed and synthesized by Shanghai Genechem Co., Ltd. (Shanghai, China). Sense and antisense primer sequences of the siRNA are reported in Table 1. MAC-T cells were seeded into 6-well plates at a density of 2.0 × 10^6^ cells/mL. The siRNA was mixed with Lipofectamine 2000 (Invitrogen, Carlsbad, CA, USA) in serum-free, antibiotic-free DMEM/F12 medium and then incubation for 20 min in room temperature. MAC-T cells were then treated with 50 nmol/L of Si-NFE2L2 or Si-Control/Lipofectamine solution in antibiotic-free DMEM/F12 medium for 12 h. Medium was then switched to DMEM/F12 medium supplemented containing 10% fetal bovine serum, 100 U/mL penicillin, and 100 μg/mL streptomycin for 36 h.

**Table 1:** Sense and antisense primer sequences of the siRNA

siRNA	Sense primer (5′ to 3′)	Antisense primer (5′ to 3′)
*Si-NFE2L2*	CTGGAGCAAGATTTAGATCAT	ATGATCTAAATCTTGCTCCAG
*Si-Control*	UUCUCCGAACGUGUCACGUdTd	ACGUGACACGUUCGGAGAAdTd

### Total RNA extraction and quantitative reverse-transcription PCR analysis

Cells were washed twice with PBS before isolating total RNA with RNAiso Plus (AJG1895A, TaKaRa Biotechnology Co. Ltd., Dalian, China) according to the manufacturer’s protocol. Concentration of total RNA was measured with a K5500 Micro Spectrophotometer (Beijing Kaiao Technology Development Ltd., Beijing, China). RNA integrity was determined by RNA integrity number with the Agilent 2100 bioanalyzer (Agilent Technologies, Santa Clara, CA, USA). All samples had an RNA integrity number above 8.0. Subsequently, 1 μg of total RNA were reverse-transcribed into cDNA according to the instructions of the reverse transcription kit (TaKaRa Biotechnology Co. Ltd., Tokyo, Japan). The mRNA abundance was measured using a SYBR Green Plus Reagent Kit (TaKaRa Biotechnology Co. Ltd.) with the 7500 Real-Time PCR System (Applied Biosystems, Foster City, CA, USA). Calculated mRNA abundance levels was via the 2^–ΔΔCT^ method, and normalized to the mean of *GAPDH* and *ubiquitin B*. Primer sequences are reported in Table 2. Results were expressed as fold changes by normalizing the data to the control values.

**Table 2:** Primer sequences of the genes analyzed

Gene	Primer sequences (5′ to 3′)	Length, bp
*NFE2L2*	F: GCCCTCACTGGATAAAGAA R: CATGCCGTTGCTGGTAC	202
*GAPDH*	F: GTCTTCACTACCATGGAGAAGG R: TCATGGATGACCTTGGCCAG	197
*Ubiquitin B*	F: AGATCCAGGATAAGGAAGGCAT R: GCTCCACCTCCAGGGTGAT	198

### Protein extraction and western blotting

Total protein was lysed by RIPA (R0020; Solarbio, Beijing, China) buffer containing PMSF (P0100; Solarbio, Beijing, China). Nuclear protein was extracted from cells with a nuclear protein extraction kit (P0013; Beyotime Institute of Biotechnology) according to protocols from the supplier. Concentrations of protein were measured by the bicinchoninic acid assay (P1511, Applygen Technologies, Beijing, China) according to the manufacturer’s protocol. Subsequently, protein samples (30 μg/lane) were separated using 10% or 12% SDS-PAGE and electro-transferred onto 0.45 μm polyvinylidene difluoride (PVDF) membranes (Millipore Corp., Billerica, MA, USA). Then, the membrane was blocked by 5% Skim Milk (9252346, BD) in Tris-buffered saline (TBST; 50 mmol/L Tris, pH 7.6, 150 mmol/L NaCl, and 0.1% Tween 20) for 4 h at room temperature. The PVDF membranes were then incubated overnight at 4 °C with specific antibodies for p62 (1:1000; ab101266, Abcam, Cambridge, MA, USA), LC3 (1:1000; ab48394, Abcam), NFE2L2 (1:1000; ab137550, Abcam), GAPDH (1:5000; abs132004; Absin (Shanghai) Biotechnology Co., Shanghai, China), histone H3 (1:1000; 4499, Cell Signaling Technology Danvers, MA). The PVDF membranes were washed by TBST before incubate with HRP-conjugated anti-mouse or anti-rabbit antibodies (3:5000; Beyotime Biotechnology) for 45 min at room temperature. Protein abundance signals were visualized by an enhanced chemiluminescence solution detection kit (ECL, Millipore, Bedford, MA, USA) using protein imager (Protein Simple, Santa Clara, CA, USA). Bands were quantified with Image Lab software (Bio-Rad Laboratories Inc.).

### Immunofluorescence staining

MAC-T cells were washed 3 times with PBS before fixing in 4% paraformaldehyde at room temperature for 30 min. After washing 3 times with PBS, the samples were incubated with proteinase K (U8805; TIANGEN, Beijing, China) for 30 s at room temperature and washed 3 times with PBS. Cells were then incubated with 0.1% Triton X-100 (T9284; Sigma-Aldrich, St. Louis, MO, USA) for 10 min at room temperature. Subsequently, MAC-T cells were washed 3 times with PBS, and incubated by rabbit primary antibody for NFE2L2 (1:1000; ab137550, Abcam, Cambridge, MA, USA) at 4 °C overnight. Cells were then washed 3 times with PBS and incubated with goat anti-rabbit IgG conjugated with cy3 (1:200; A0516, Beyotime Institute of Biotechnology, Jiangsu, China) for 1 h at room temperature. After washing 3 times with PBS, cells were stained with DAPI (10 μg/mL) (D8417, Sigma-Aldrich) at room temperature for 10 min. After washing 3 times with PBS, the image was collected with a laser confocal microscope (Fluoview FV1200, Olympus, Tokyo, Japan).

### Determination of intracellular ROS

After treating as indicated above, cells were detached with trypsin and incubated at 37 °C for 5 min before collecting into centrifuge tubes. Cells were washed 3 times with PBS and incubated with 25 μmol/L 2′,7′-dichlorofluorescein diacetate (Beyotime Institute of Biotechnology) in serum-free DMEM/F12 medium at 37 °C for 20 min in the dark. Cells were then washed 3 times with PBS before resuspending with bench-top solution. Subsequently, fluorescence of cells was determined using flow cytometry (FACSCalibur, Becton-Dickinson, Sunnyvale, CA, USA).

### Detection of oxidative stress indicators

Activities of superoxide dismutase (SOD), glutathione peroxidase (GSH-Px), and catalase (CAT), and content of malondialdehyde (MDA) in MAC-T cells were measured by commercially available kits according to the manufacturer’s protocol as previous described [24].

### Statistical analysis

All experiments were repeated at least 3 times on different days. The data were analyzed using SPSS 23.0 software (IBM Corp., Armonk, NY) and results are presented as the means ± SEM. The Shapiro-Wilk and Levene tests were used to test the normality and variance homogeneity of data. Linear and quadratic comparisons were conducted to evaluate dose-dependent effects. Statistical significance was evaluated by paired *t*-test between the 2 groups (Exp. 3) via one-way ANOVA among the 4 groups (Exp. 1, 2 and 4) followed by Bonferroni correction. Differences with *P* < 0.05 were considered statistically significant.

## Results

### FFA induced oxidative stress in MAC-T cells

**Fig. 1:**
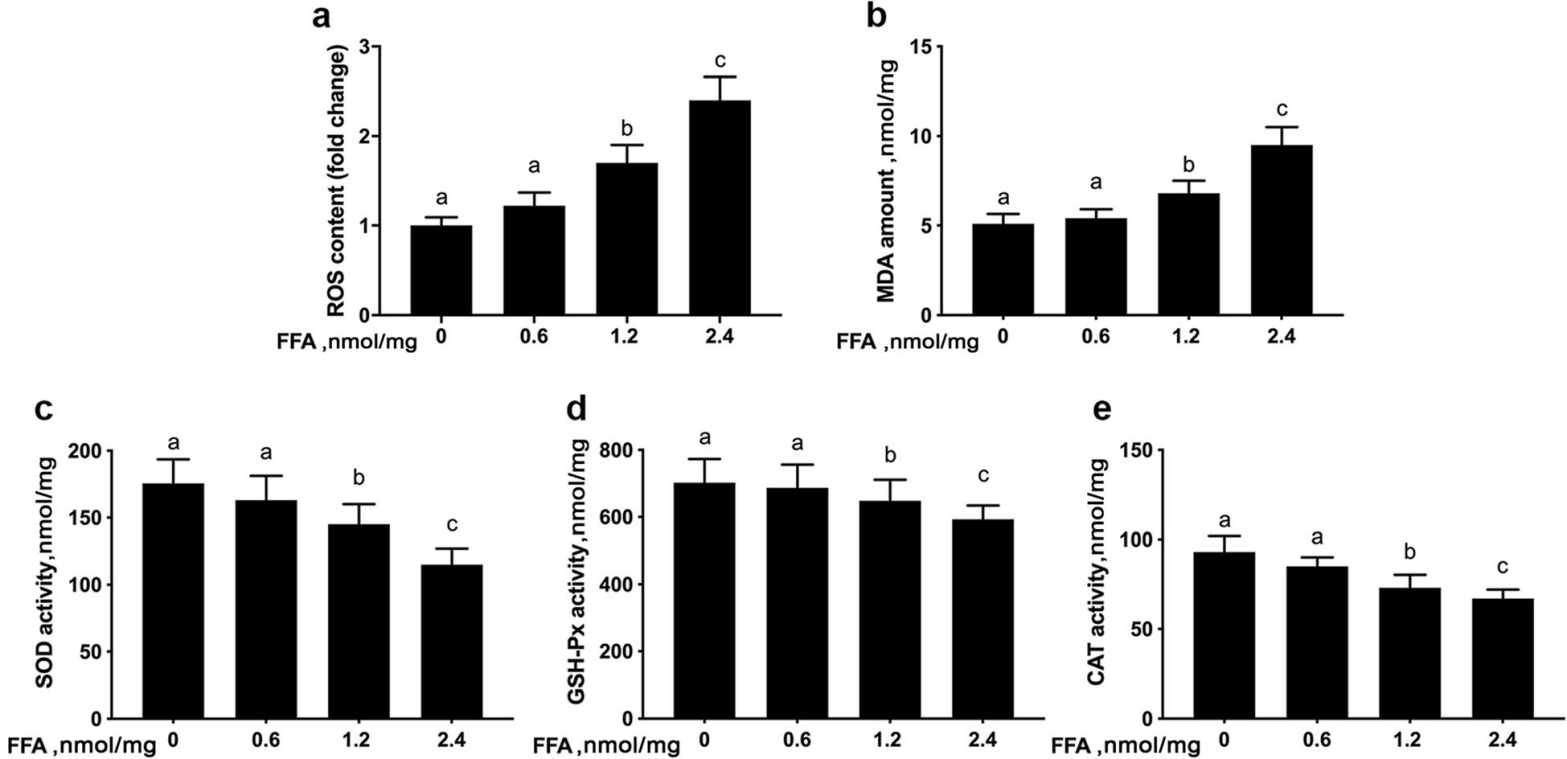
FFA induced oxidative stress in MAC-T cells. MAC-T cells were treated with 0, 0.6, 1.2 or 2.4 mmol/L FFA for 24 h. **a** ROS content. **b** MDA content. **c-e** Activities of SOD, GSH-Px and CAT. Comparisons among groups were calculated using a one-way ANOVA with subsequent Bonferroni correction. The data presented are the mean ± SEM. Different superscript lowercase letters in bar charts represent significant difference (*P* < 0.05)

Compared with 0 mmol/L FFA, the level of intracellular ROS (linear, *P* < 0.001, Fig. 1a and Table 3) and the content of MDA (linear and quadratic, *P* < 0.001, Fig. 1b and Table 3) was greater with 1.2 or 2.4 mmol/L FFA. Furthermore, compared with 0 mmol/L FFA, activities of SOD (linear, *P* < 0.001, Fig. 1c and Table 3), GSH-Px (linear and quadratic, *P* < 0.001, Fig. 1d and Table 3), and CAT (linear and quadratic, *P* < 0.001, Fig. 1e and Table 3) were lower with 1.2 and 2.4 mmol/L FFA.

**Table 3:** Linear and quadratic contrasts in MAC-T cells incubated with increasing concentrations of FFA

Item	SEM	*P*	
		Linear	Quadratic
ROS	0.080	< 0.001	0.604
MDA	0.086	< 0.001	< 0.001
SOD	6.293	< 0.001	0.351
GSH-Px	1.000	< 0.001	< 0.001
CAT	0.573	< 0.001	< 0.001
LC3-II	0.059	< 0.001	0.005
p62	0.079	< 0.001	0.046
NFE2L2	0.047	< 0.001	< 0.001

*ROS* Reactive oxygen species, *MDA* Malondialdehyde, *SOD* Superoxide dismutase, *GSH-Px* Glutathione peroxidase, *CAT* Catalase, *LC3-II* Microtubule-associated protein 1A/1B-light chain 3-II, NFE2L2 = Nuclear factor erythroid 2 related factor 2

### FFA inhibit autophagy in MAC-T cells

**Fig. 2:**
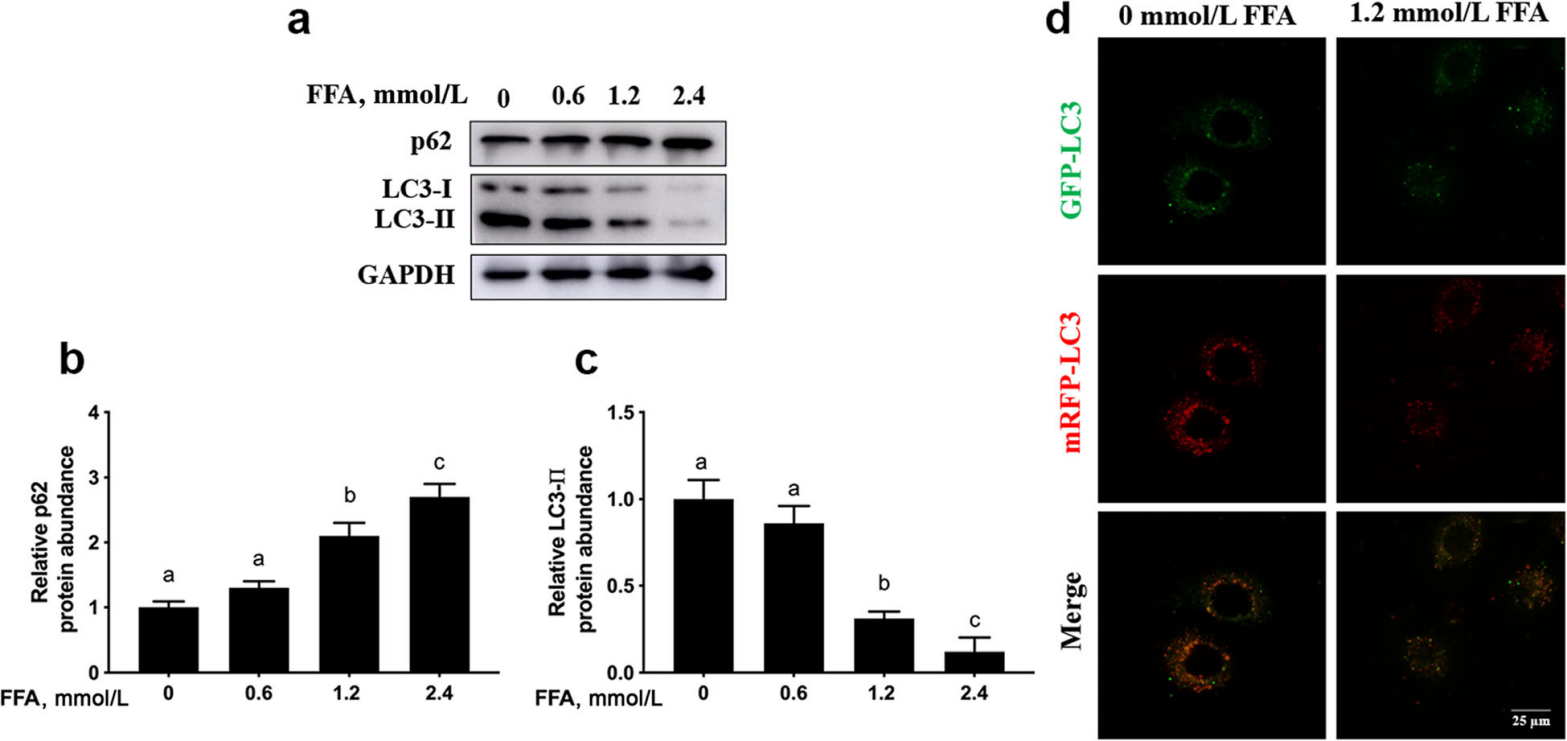
FFA inhibited autophagy in MAC-T cells. MAC-T cells were treated with 0, 0.6, 1.2 or 2.4 mmol/L FFA for 24 h. **a** Western blot analysis of p62 and LC3-II. **b** Protein abundance of p62. **c** Protein abundance of LC3-II. **d** MAC-T cells were transfected with the recombinant adenovirus mRFP-GFP-LC3 for 48 h, and/or treated with 1.2 mmol/L FFA for another 24 h after serum-starvation for 12 h. Representative images of autophagosomes (yellow puncta) and autolysosomes (red puncta), scale bar = 25 μm. Comparisons among groups were calculated using a one-way ANOVA with subsequent Bonferroni correction. The data presented are the mean ± SEM. Different superscript lowercase letters in bar charts represent significant difference (*P* < 0.05)

Compared with 0 mmol/L FFA, protein abundance of p62 was greater in the 1.2 and 2.4 mmol/L FFA group (linear, *P* < 0.001, Fig. 2a and b and Table 3). In contrast, compared with 0 mmol/L FFA, protein abundance of LC3-II was lower with 1.2 and 2.4 mmol/L FFA (linear, *P* < 0.001, Fig. 2a and c and Table 3). Compared with 0 mmol/L FFA, the number of autophagosomes labeled with yellow puncta and autolysosomes labeled with red puncta was greater with 1.2 mmol/L FFA group (Fig. 2d).

### Activation of autophagy attenuated FFA-induced ROS accumulation

**Fig. 3:**
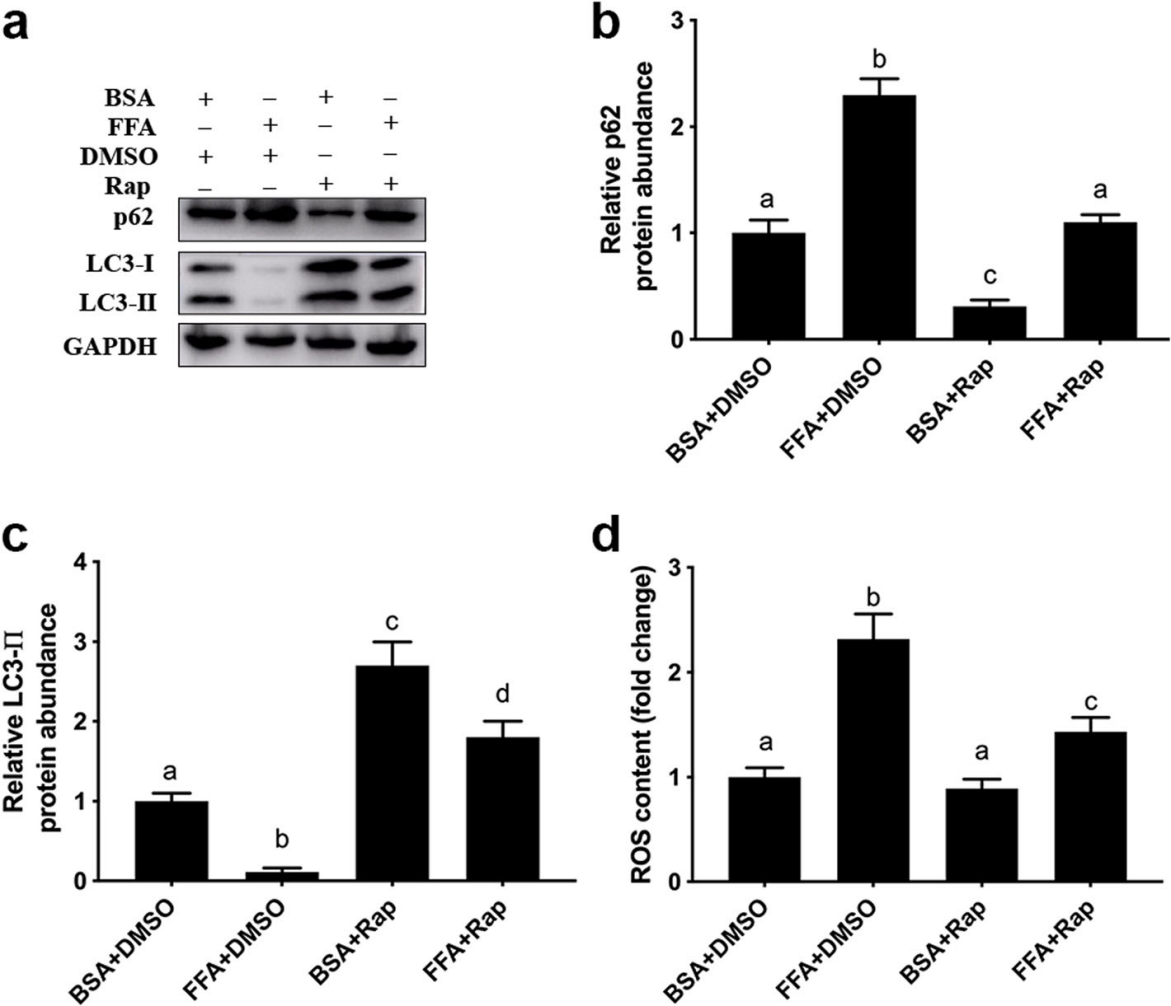
Activation of autophagy attenuated FFA-induced ROS accumulation in MAC-T cells. There were 4 experimental treatments: BSA + DMSO group, DMSO were used to treat cells for 18 h prior to use 2% BSA treating cells for an additional 24 h; 1.2 mmol/L FFA + DMSO group, DMSO were used to treat cells for 18 h before 1.2 mmol/L FFA treating cells for 24 h; BSA + 100 nmol/L Rap group, 100 nmol/L Rap were used to treat cells for 18 h followed by 2% BSA treating cells for an additional 24 h; FFA + 100 nmol/L Rap group, 100 nmol/L Rap were used to treat cells for 18 h followed by 1.2 mmol/L FFA treating cells for an additional 24 h. **a** Western blot analysis of LC3-II and p62. **b** Protein abundance of p62. **c** Protein abundance of LC3-II. **d** ROS content. Comparisons among groups were calculated using a one-way ANOVA with subsequent Bonferroni correction. The data presented are the mean ± SEM. Different superscript lowercase letters in bar charts represent significant difference (*P* < 0.05)

Compared with BSA + DMSO, protein abundance of p62 was greater with 1.2 mmol/L FFA + DMSO group (*P* < 0.001, Fig. 3a and b). However, pretreatment with 100 nmol/L Rap, an activator of autophagy, decreased protein abundance of p62 (*P* = 0.001) and attenuated the upregulation of protein abundance of p62 induced by FFA (*P* < 0.001, Fig. 3a and b). Pretreatment with 100 nmol/L Rap increased protein abundance of LC3-II (*P* < 0.001) and attenuated the downregulation of protein abundance of LC3-II induced by FFA (*P* < 0.001, Fig. 3a and c). Furthermore, compared with 1.2 mmol/L FFA + DMSO, ROS content was lower with 1.2 mmol/L FFA + 100 nmol/L Rap (*P* = 0.003, Fig. 3d).

### FFA inhibit translocation of NNFE2L2 into the nucleus in MAC-T cells

**Fig. 4:**
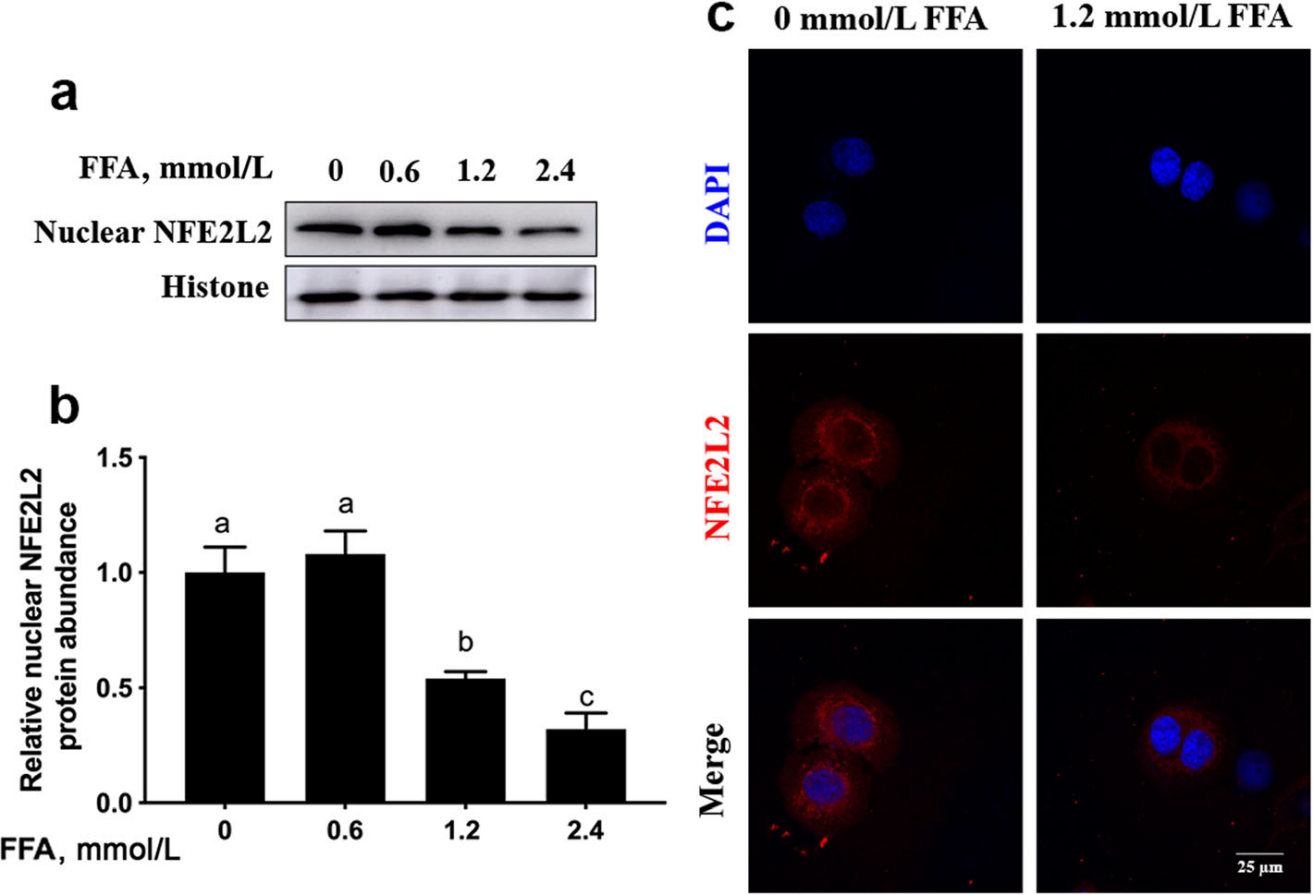
FFA inhibited translocation of NFE2L2 into the nucleus in MAC-T cells. MAC-T cells were treated with 0, 0.6, 1.2 or 2.4 mmol/L FFA for 24 h. **a** Western blot analysis of nuclear NFE2L2 in MAC-T cells. **b** Protein abundance of nuclear NFFE2L2. **c** MAC-T cells were treated with 1.2 mmol/L FFA for 24 h. Immunofluorescence for NFE2L2 (red) was performed, and the nuclear dye DAPI (blue) was used, scale bar = 25 μm. Comparisons among groups were calculated using a one-way ANOVA with subsequent Bonferroni correction. The data presented are the mean ± SEM. Different superscript lowercase letters in bar charts represent significant difference (*P* < 0.05)

Compared with 0 mmol/L FFA, nuclear protein abundance of NFE2L2 was lower with 1.2 or 2.4 mmol/L FFA (linear and quadratic, *P* < 0.001, Fig. 4a and b and Table 3). Consistent with alterations in nuclear protein abundance of NFE2L2, immunofluorescence staining results revealed that FFA treatment inhibited NFE2L2 translocation to the nucleus (Fig. 4c).

### Knockdown of NFE2L2 inhibit autophagy in MAC-T cells

**Fig. 5:**
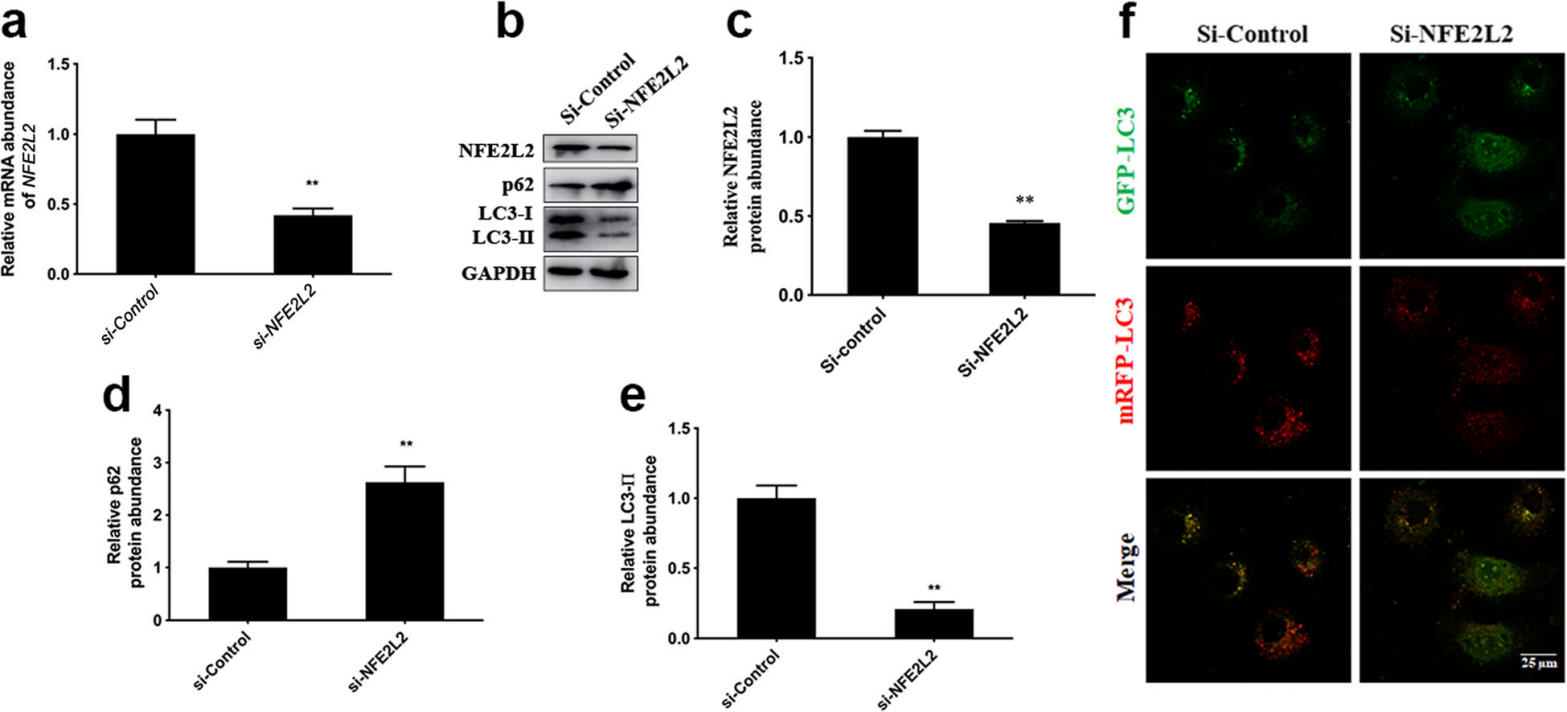
Knockdown of *NFE2L2* inhibited autophagy in MAC-T cells. Scrambled non-target negative control (Si-Control) or siRNA targeting NFE2L2 (Si-NFE2L2) were used to transfect in MAC-T cells for 48 h. **a** Relative mRNA abundance of *NFE2L2*. **b** Western blot analysis of NFE2L2, LC3-II and p62. **c** Protein abundance of NFE2L2. **d** Protein abundance of p62. **e** Protein abundance of LC3-II. **f** MAC-T cells were co-transfected with the recombinant adenovirus mRFP-GFP-LC3 and NFE2L2 siRNA for 48 h. Representative images of autophagosomes (yellow puncta) and autolysosomes (red puncta), scale bar = 25 μm. Data were analyzed with paired *t*-test. The data presented are the mean ± SEM. ** *P* < 0.01

Compared with the Si-Control, mRNA abundance of *NFE2L2* (*P* = 0.006, Fig. 5a) and protein abundance of NFE2L2 was lower in Si-NFE2L2 (*P* < 0.001, Fig. 5b and c). Compared with Si-Control, protein abundance of p62 was greater in Si-NFE2L2 (*P* < 0.001, Fig. 5b and d). In contrast, compared with Si-Control, protein abundance of LC3-II was lower in Si-NFE2L2 (*P* < 0.001, Fig. 5b and e). Consistent with alterations in protein abundance of LC3-II, knockdown of *NFE2L2* decreased the number of autophagosomes labeled with yellow puncta and autolysosomes labeled with red puncta (Fig. 5f).

### Activation of NFE2L2 attenuated FFA-induced autophagy inhibition and oxidative stress

**Fig. 6:**
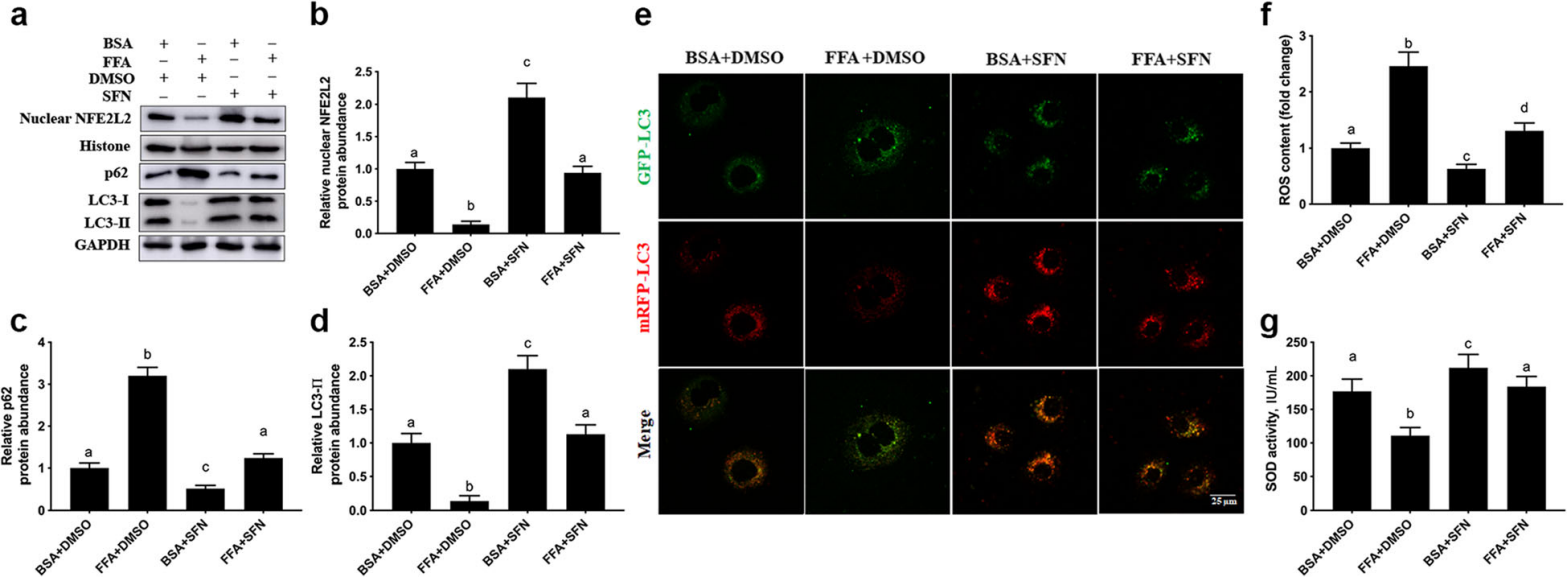
Activation of NFE2L2 attenuated the inhibition of autophagy and oxidative stress induced by FFA. The 4 experimental treatments included the following: BSA + DMSO group, DMSO were used to treat cells for 24 h prior to use 2% BSA treating cells for an additional 24 h; 1.2 mmol/L FFA + DMSO group, DMSO were used to treat cells for 24 h before 1.2 mmol/L FFA treating cells for 24 h; BSA + 10 μmol/L SFN group, 10 μmol/L SFN were used to treat cells for 24 h followed by 2% BSA treating cells for an additional 24 h; FFA + 10 μmol/L SFN group, 10 μmol/L SFN were used to treat cells for 24 h followed by 1.2 mmol/L FFA treating cells for an additional 24 h. **a** Western blot analysis of NFE2L2, p62 and LC3-II. **b** Protein abundance of NFE2L2. **c** Protein abundance of p62. **d** Protein abundance of LC3-II. **e** MAC-T cells were transfected with the recombinant adenovirus mRFP-GFP-LC3 for 36 h and with/without SFN for another 12 h, before treated with/without 1.2 mmol/L FFA for another 24 h. Representative images of autophagosomes (yellow puncta) and autolysosomes (red puncta), scale bar = 25 μm. **f** ROS content. **g** SOD activity. Comparisons among groups were calculated using a one-way ANOVA with subsequent Duncan correction. The data presented are the mean ± SEM. Different superscript lowercase letters in bar charts represent significant difference (*P* < 0.05)

Compared with BSA + DMSO, protein abundance of nuclear NFE2L2 was lower with 1.2 mmol/L FFA + DMSO (*P* = 0.001, Fig. 6a and b). However, pretreatment with 10 μmol/L SFN, an activator of NFE2L2, increased protein abundance of nuclear NFE2L2 (*P* < 0.001) and attenuated the down-regulation of protein abundance of nuclear NFE2L2 induced by FFA (*P* = 0.002, Fig. 6a and b). Pretreatment with 10 μmol/L SFN decreased protein abundance of p62 (*P* = 0.034) and attenuated the upregulation of protein abundance of p62 induced by FFA (*P* < 0.001, Fig. 6a and c), while it increased protein abundance of LC3-II (*P* < 0.001, Fig. 6a and d) and attenuated the down-regulation of protein abundance of LC3-II induced by FFA (*P* < 0.001, Fig. 6a and d). Consistent with alterations in protein abundance of LC3-II, the number of autophagosomes labeled with yellow puncta and autolysosomes labeled with red puncta were greater with 1.2 mmol/L FFA + 10 μmol/L SFN (Fig. 6e). Pretreatment with 10 μmol/L SFN decreased content of ROS (*P* = 0.042, Fig. 6f) and attenuated the increase in content of ROS induced by FFA (*P* < 0.001, Fig. 6f), while it decreased the activity of SOD and attenuated the increase in activity of SOD induced by FFA (*P* = 0.032, Fig. 6g).

## Discussion

The concentrations of FFA in plasma of dairy cows after calving increase due to severe negative energy balance [25], and can induce oxidative stress in the mammary gland [3]. Work with vitro experiments has confirmed that exogenous FFA induce ROS production in bovine mammary epithelial cells, resulting in oxidative stress [6]. Thus, the greater ROS and MDA content when cells were challenged with FFA in the present study underscored the positive effect of this metabolite on free radical production and levels of lipid peroxidation products. In fact, greater ROS and MDA content coupled with lower activities of SOD, GSH-Px, and CAT confirmed that exogenous FFA induced oxidative stress in mammary epithelial cells in a dose-dependent manner. Thus, we speculated that mammary uptake of FFA when circulating concentrations increase markedly is conducive to a state of oxidative stress that could impact cell function. In fact, the degree of oxidative stress in mammary tissue of ketotic cows was correlated positively with the circulating concentrations of FFA [26].

Autophagy serves to reduce oxidative stress through removal of protein aggregates and damaged organelles [27, 28]. The microtubule-associated protein light chain 3 (LC3) is processed from the cytosolic form (LC3-I) to the LC3-II during autophagy, the protein abundance of which is positively correlated with autophagic activity [29, 30]. The protein p62 is an autophagy receptor involved in the recognition of ubiquitin-labeled substrates targeted for autophagy, the protein abundance of which is inversely correlated with autophagic activity [31]. In the present study, the lower protein abundance of LC3-II along with greater protein abundance of p62 underscored the negative effect of FFA on autophagy in mammary cells. Work with mice has demonstrated that high circulating concentrations of FFA due to metabolic stress impair autophagy and leads to oxidative stress [32]. Thus, inhibition of autophagy due to metabolic stress might induce oxidative stress in bovine mammary epithelial cells.

During autophagy, cellular contents are engulfed by double-membrane vesicles called autophagosomes and subsequently delivered to lysosomes for degradation [33]. Thus, down-regulation of autophagosomes (yellow puncta) and autolysosomes (red puncta) further indicated that FFA inhibited autophagic flux in mammary epithelial cells. Work by Sun et al. [4] reported that activation of autophagy alleviated H_2_O_2_-induced oxidative stress in bovine mammary epithelial cells. In non-ruminants, Rap, an activator of autophagy, can enhance autophagy through upregulation of LC3-II and downregulation of p62, and promotion of fusion between the autophagosomes and lysosomes [34, 35]. In this study, the increased protein abundance of LC3-II and the decreased protein abundance of p62 confirmed the role of Rap on autophagy in mammary epithelial cells. The decrease in intracellular ROS levels due to autophagy activation in the present study agreed with previous work demonstrating a cytoprotective role of autophagy against oxidative stress [36].

At least in non-ruminants, the transcription factor NFE2L2, besides its role as a master regulator of cellular homeostasis, plays important roles in autophagy [13, 37]. In the present study, the decrease in nuclear protein abundance of NFE2L2 in response to FFA treatment was consistent with alterations in autophagy, and agrees with similar work in HepG2 cells and bovine hepatocytes [38, 39]. Thus, we speculate that alterations in protein abundance of NFE2L2 are closely related to inhibition of autophagy induced by metabolic stress.

Knockdown of NFE2L2 deceased LC3-II protein abundance in PC12 cells [40]. In agreement with those data, the fact that knockdown of NFE2L2 via siRNA increased protein abundance of p62 and decreased both protein abundance of LC3-II and number of autophagosomes and autolysosomes suggested that NFE2L2 directly regulated autophagy. Thus, promoting NFE2L2 translocation into the nucleus in mammary cells to activate the autophagy pathway may be a potential treatment strategy for oxidative stress.

The specific mechanisms whereby FFA inhibit NFE2L2 transport to the nucleus in bovine mammary epithelial cells remains to be elucidated. From a regulatory standpoint, it is noteworthy that Keap1, a component of the E3 ubiquitin ligase complex, can directly regulate the abundance and nuclear translocation of NFE2L2 [41]. We speculated that decreased nuclear translocation of NFE2L2 was partly due to inhibition of the detachment of Keap1-NFE2L2. In fact, previous research confirmed that protein abundance of Keap1 is reduced in adipose tissue of dairy cows with ketosis [42].

Enhanced autophagy is thought to be a response to mitigate oxidative stress. NFE2L2 modulated autophagic flux in addition to upregulating antioxidant defenses via the p62-NFE2L2 feedback pathway [43]. Activation of NFE2L2 by SFN increased autophagy activity via inducing LC3-II elevation and vesicle formation in lens cells [44]. In the present study, the upregulation of LC3-II protein abundance and downregulation of p62 protein abundance in response to activation of NFE2L2 indicated that it plays a similar role in bovine mammary epithelial cells in promoting autophagic flux. Furthermore, the downregulation of protein abundance of LC3-II, decrease in number of autophagosomes and autolysosomes, decrease in intracellular ROS, and the increase of SOD activity in FFA-challenged cells in which NFE2L2 was activated with SFN underscored the positive effect of NFE2L2-mediated autophagy on oxidative stress. Thus, we speculate that autophagy was activated through NFE2L2 translocation into the nucleus to alleviate oxidative stress. Our data validated previous work demonstrating that activation of the NFE2L2 pathway upregulates autophagy [45], and activation of autophagy is a regulatory mechanism to attenuate oxidative stress [46].

## Conclusions

The present findings indicated that FFA induces oxidative stress and inhibits NFE2L2-mediated autophagy in bovine mammary epithelial cells. Activation of NFE2L2-mediated autophagy via SFN alleviated FFA-induced oxidative stress. Taken together, the present study confirmed that NFE2L2-mediated autophagy may be a promising therapeutic target for reducing FFA-induced oxidative stress of bovine mammary gland during periods such as the transition into lactation.

## Data Availability

All data generated or analyzed during this study are included in this published article.

## Abbreviations

NFE2L2: Nuclear factor erythroid 2 related factor 2
FFA: Free fatty acids
MAC-T: Bovine mammary epithelial cells
siRNA: Small interfering RNA
SFN: Sulforaphane
GSH-Px: Glutathione peroxidase
CAT: Catalase
SOD: Superoxide dismutase
ROS: Reactive oxygen species
MDA: Malondialdehyde
LC3-II: LC3-phosphatidylethanolamine conjugate
Rap: Rapamycin
Si-NFE2L2: NFE2L2 small interfering RNA
DMSO: Dimethylsulf oxide
PBS: Phosphate-buffered saline
Si-Control: Scrambled non-target negative control
PVDF: Polyvinylidene difluoride
LC3: Microtubule-associated protein light chain 3
DMEM/F-12: Dulbecco’s modified eagle’s medium: nutrient mixture F-12 medium
DAPI: 4′,6-diamidino-2-phenylindole dihydrochloride
TBST: Tris-buffered saline
ECL: Enhanced chemiluminescence solution
